# Aryl hydrocarbon receptor signaling involves in the human intestinal ILC3/ILC1 conversion in the inflamed terminal ileum of Crohn’s disease patients

**DOI:** 10.14800/ics.1404

**Published:** 2016-08-29

**Authors:** Jian Li, Andria Doty, Sarah C. Glover

**Affiliations:** Division of Gastroenterology, Hepatology and Nutrition, Department of Medicine, University of Florida P.O. Box 100214, Gainesville, FL, 32610 USA

**Keywords:** ILC, AHR, CD, terminal ileum

## Abstract

Innate lymphoid cells (ILCs) are emerging as important components of our immune system that have critical effector and regulatory functions in both innate and adaptive immune responses. They are enriched at mucosal surfaces, such as lung and intestine. Our previous work has shown that Lineage^−^CRTH2^−^CD45^+^NKp44^−^CD117^−^CD127^+^ILC1s accumulated in the inflamed terminal ileum of patients with Crohn’s disease (CD) at the expense of NKp44^+^ILC3s. This phenotype conversion impairs the intestinal barrier integrity and contributes to the dysregulated immune responses of CD patients. Our next step was to search for pathways to modulate this phenotype switch. The aryl hydrocarbon receptor (AHR) is a ligand-dependent transcription factor. Initial studies of AHR concentrated on its role in the detoxification of xenobiotics. However, recent research has focused on the immune system. Especially, AHR pathway is proven to be essential for the maintenance of intestinal ILC3s in mouse models. We examined whether AHR pathway participated in the human intestinal ILC phenotype change in the inflamed terminal ileum of CD patients. As anticipated, NKp44^+^ILC3s, NKp44^−^ILC3s and ILC1s had differential AHR expression. This AHR signaling mediated CD117 expression on the surface of ILC3s. The conversion from ILC3 to ILC1 was accompanied by the downregulation of AHR expression. We further observed that there was a disparity between AHR protein expression and mRNA expression in the inflamed terminal ileum tissues of CD patients compared to unaffected areas. These findings suggest that AHR pathway is also important for human intestinal ILC phenotype regulation and impaired AHR signaling in the inflamed gut of CD patients possibly contributes to the ILC3/ILC1 conversion.

CD is a chronic inflammatory condition of the gastrointestinal tract and may affect any part from the mouth to the anus. The exact cause of CD remains unknown. It has been suggested that an abnormal immune response against the microorganisms within the gut is responsible for the disease in genetically susceptible individuals ^[1, 2]^. Recently, a rapid and exciting expansion in our knowledge of the mucosal immunity has occurred. An example is the identification of new cell types, such as ILCs.

ILCs are the first line of defense in our immune system to respond to danger signals from the environment by rapidly secreting cytokines and chemokines. They are categorized into three groups: ILC1, ILC2 and ILC3 according to their cytokines production and transcription factors profile ^[3–6]^. In mice, ILC3s play a crucial role in gut immunity, by directly inducing epithelial cell proliferation and glycosylation, promoting anti-microbial peptides production, preventing dissemination of commensal bacteria, and limiting commensal bacteria specific CD4^+^ T cell response ^[7–11]^. In human, NKp44^+^ILC3s have been shown to be the major ILC subset in the intestine at the steady state ^[12]^. In the context of CD, our group and other groups have shown that there were significant changes regarding ILCs composition in the inflamed gut. NKp44^+^ILC3s were found to be significantly reduced while IFN-γ-producing ILC1s accumulated in the inflamed intestinal mucosa of CD patients compared to non-inflamed areas ^[13–20]^. This phenotype alteration switches ILCs’ role from protective to pathogenic in the maintenance of human intestinal immune homeostasis.

AHR belongs to the family of basic helix-loop-helix/Per-Arnt-Sim (bHLH/PAS) proteins. It is a ligand-dependent transcription factor located in the cytoplasm of cells that upon ligand binding translocates to the nucleus, dimerizes with its nuclear translocator (ARNT), and binds to AHR response elements in the promoter regions of its target genes to initiate their transcription ^[21–23]^. Initial studies of AHR focused on its role in mediating toxicity of dioxin-like chemicals. Actually, AHR exerts multiple functions in our body, acting as an essential sensor for environmental factors and human lifestyle factors, such as diet, smoking and psychological stress changes. It is a link between environment and immunity ^[24–26]^.

Recently, accumulating evidence has indicated that AHR signaling is involved in the regulation of differentiation, maintenance and function of various mucosal immune cell subsets in mouse models, including Th17 cells, T_reg_ cells and natural killer cells ^[27–30]^. Also, AHR pathway is necessary for the maintenance and function of IL-22-producing ILC3s in the gut. AHR knock-out mice have significantly reduced numbers of IL-22-producing ILC3s subset in the gut, lack of lymphoid tissues cryptopatches and isolated lymphoid follicles and less IL-22 production ^[31–33]^. Interestingly, AHR expression has been shown to be down-regulated in the inflamed mucosa of CD patients ^[34, 35]^. However, the association between AHR expression and ILC3s population in the human gastrointestinal tract remains unclear.

Based on all these information, we hypothesized that NKp44^+^ILC3s subset played a protective role in the pathogenesis of CD and AHR signaling was essential for the maintenance of this subset in the intestinal mucosal lamina propria of CD patients. We found that the three intestinal ILC subsets (NKp44^+^ILC3, NKp44^−^ILC3 and ILC1) had differential AHR expression levels. AHR expression in the NKp44^+^ILC3s was the highest while its expression in the NKp44^−^ILC3s was intermediate. ILC1s did not express AHR at all. This means that during the ILC3/ILC1 conversion process, the AHR expression in the NKp44^+^ILC3s needs to be downregulated. Our data proved that the AHR expression was down-regulated in the inflamed NKp44^+^ILC3s compared to ILC3s from unaffected tissues. Also, we noticed that there was a disparity between AHR protein expression and mRNA expression in the inflamed terminal ileum of our CD patients. AHR protein expression was downregulated while its mRNA expression was upregulated. It is likely that AHR expression in the inflamed gut of CD patients is regulated by a post-transcriptional mechanism. This is consistent with a recent study which suggests that MicroRNA-124 targets AHR in CD to promote intestinal inflammation ^[35]^.

The impaired AHR signaling pathway causes the NKp44^+^ILC3s phenotypic instability in the inflamed gut of CD patients. The potential mechanism probably involves the stable expression of surface marker CD117 which is essential for ILCs phenotype ^[36]^. In mouse models, one group demonstrates that intestinal ILC3s of their AHR knock-out mice model has significantly reduced expression CD117 ^[31]^. Their chromatin immunoprecipitation (ChIP) study further suggests that two canonical AHR binding elements are located in the promoter of CD117 gene ^[31]^. In addition, another two groups have shown that CD117 is under the direct transcriptional control of AHR ^[37, 38]^. For human innate lymphoid cells, AHR pathway can regulate their CD117 expression as well ^[39, 40]^. We also observed that CD117 expression was downregulated in the inflamed NKp44^+^ILC3s. This AHR mediated CD117 expression contributes to the maintenance of NKp44^+^ILC3s phenotype in the human gut.

In summary, our findings suggest that impaired AHR signaling involves in the human intestinal ILC3/ILC1 conversion in the inflamed terminal ileum of CD patients and could be a new target of immunotherapy for those patients.
